# Efflux systems driving resistance and virulence across biological domains

**DOI:** 10.7717/peerj.20360

**Published:** 2025-11-26

**Authors:** Pedro Eduardo Almeida da Silva, Andrea Von Groll, Ivy Ramis, Ana Julia Reis, Daniela Ramos, Miguel Viveiros

**Affiliations:** 1 Universidade Federal do Rio Grande, Rio Grande, RS, Brazil; 2 Instituto de Higiene e Medicina Tropical, Universidade Nova de Lisboa, Lisboa, Portugal

**Keywords:** Efflux pumps, Multidrug resistance, Virulence factors, Efflux inhibitors, Therapeutic strategies

## Abstract

**Background:**

Efflux pumps (EPs) are key contributors to multidrug resistance (MDR) in bacteria, fungi, and cancer cells. These membrane proteins actively extrude a variety of therapeutic agents, reducing their intracellular concentration and thus compromising the efficacy of treatment. Beyond resistance, EPs are also involved in virulence, biofilm formation, immune evasion, and environmental persistence.

**Aim:**

This review aimed to provide a comprehensive and critical synthesis of the role of efflux pumps in antimicrobial and antitumoral resistance, as well as their contribution to virulence and persistence across biological domains.

**Methodology:**

A narrative review was conducted following a structured search strategy in PubMed, Scopus, and Web of Science using combinations of terms related to efflux systems, efflux pumps, resistance mechanisms, virulence factors, detection methods, and inhibitors. The review integrates data from *in vitro*, *in silico*, and clinical studies, including both classical detection strategies and emerging technologies such as clustered regularly interspaced short palindromic repeats (CRISPR)-based modulation, biosensors, and microfluidics.

**Results:**

Efflux pumps from different families (*e.g.*, resistance-nodulation-division (RND), ATP-binding cassette (ABC), major facilitator superfamily (MFS)) are implicated in the active extrusion of antimicrobial agents, facilitating MDR and treatment failure in pathogens such as *E. coli*, *P. aeruginosa*, *M. tuberculosis*, *Candida albicans*, and cancer cells. EPs also regulate biofilm formation, virulence factor secretion, and metabolic adaptation. Classical methods for detecting efflux (*e.g.*, minimum inhibitory concentration (MIC) shifts with inhibitors, fluorometric assays) have technical limitations, while novel technologies offer improved precision. Several natural and synthetic efflux pump inhibitors (EPIs) have demonstrated efficacy in preclinical studies, yet few have progressed to clinical use due to toxicity and pharmacokinetic barriers. CRISPR interference systems and combinatory therapies represent promising advances in overcoming EP-mediated resistance.

**Conclusion:**

Efflux systems are central players in both drug resistance and pathogenicity. Although the development of effective EIs remains challenging, advances in molecular detection, gene editing, and drug design hold potential for translational breakthroughs. A deeper understanding of efflux dynamics across organisms is essential to develop adjuvant therapies and reduce the clinical impact of MDR.

## Introduction

The role of efflux pump (EP) in multidrug resistance (MDR) was first recognized in the 1970s, when P-glycoprotein (P-gp) was identified as the first ATP-binding cassette (ABC) transporter associated with drug resistance in cancer cells (Juliano & Ling, 1976). Since then, extensive research has revealed how efflux contributes to treatment failure by actively extruding a broad range of compounds—such as antimicrobials and antitumoral agents from the intracellular environment. This activity decreases drug accumulation at the target site, undermining therapeutic efficacy and fostering MDR in both microorganisms and cancer cells (Piddock, 2006; Blair et al., 2015; Darby, Callaghan & McMahon, 2011).

Beyond resistance, efflux also plays a central role in enhancing microbial virulence. By exporting toxic molecules, including host-derived antimicrobials, these systems allow pathogens to evade immune defenses and establish infections (Martinez et al., 2009). Given their involvement in both resistance and virulence, efflux has become an attractive target for adjunctive therapies (Lomovskaya et al., 2001; Venter et al., 2015).

However, the development of efflux inhibitors (EIs) has faced persistent challenges. Despite several decades of effort, only a few EIs have entered clinical trials, and none have been successfully integrated into routine therapy. Issues related to specificity, cytotoxicity, limited spectrum, and the emergence of compensatory mechanisms have restricted their translational success. These limitations highlight the urgent need for novel inhibitors and alternative strategies to address efflux-mediated resistance.

A major barrier to advancing this field lies in the difficulty of detecting and quantifying efflux activity in clinical and laboratory settings. Standard antimicrobial susceptibility tests—such as disk diffusion and broth microdilution often fail to differentiate efflux-mediated resistance from other mechanisms (Kaatz, McAleese & Seo, 2005; Piddock, 2006). Although tools such as fluorometric assays, molecular approaches, and whole-genome sequencing have improved our understanding, their high cost, technical complexity, or limited applicability, constrain their widespread use and may compromise the accuracy of the results (Martins et al., 2011; Villellas et al., 2017). Recently, emerging technologies have shown promise in improving detection accuracy (Kim et al., 2021a).

This review provides a comprehensive overview of efflux mechanism, their role in drug resistance, and their contribution to microbial virulence. We discuss the classification, mechanisms, and therapeutic implications of EPs, as well as the methodological challenges in their detection. Finally, we explore current advances in EI and propose future directions for overcoming this complex resistance mechanism (Nikaido, 2011; Poole, 2007; Silva Jr et al., 2017; Pages, Amaral & Fanning, 2011).

## Methodology

**Figure 1:**
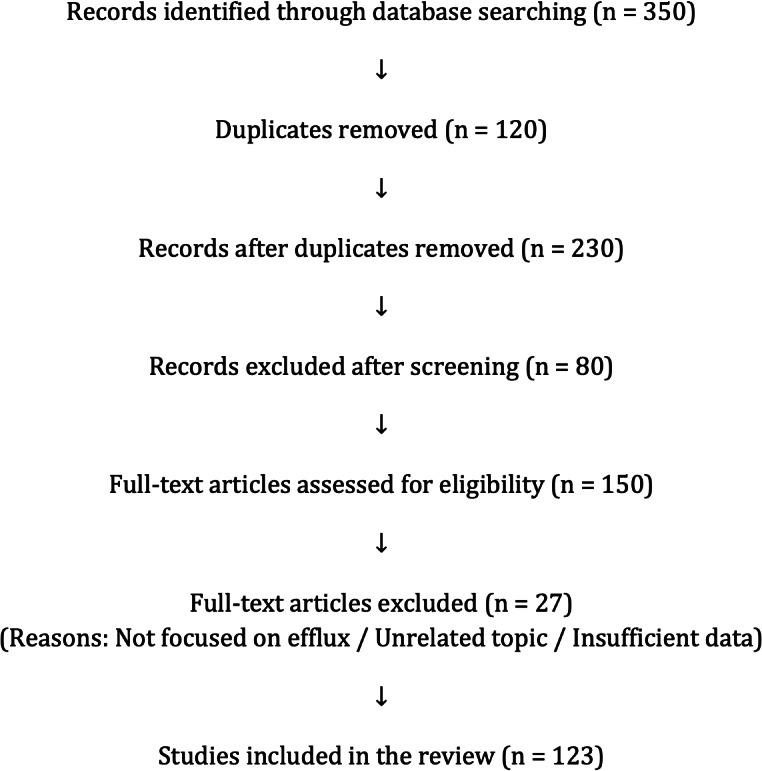
PRISMA flow diagram. Study selection process for this narrative review. From 350 records initially identified, 123 studies were included after screening, eligibility assessment, and full-text evaluation.

This narrative review presents a critical analysis of peer-reviewed literature on the role of EPs in antimicrobial and antitumoral resistance, virulence, and environmental adaptation. A comprehensive search was conducted in PubMed, Scopus, and Web of Science using combinations of keywords such as “efflux pumps,” “antimicrobial resistance,” “virulence,” “biofilms,” “EP inhibitors,” and “drug resistance in cancer.” The selection prioritized recent publications (last 15 years), along with seminal studies relevant to efflux mechanisms, detection, and therapeutic strategies in bacteria, fungi, and cancer cells. Data extraction focused on EP mechanisms, treatment impact, detection tools, biofilm involvement, and the development of EIs. The review integrates findings from *in vitro* studies, *in silico* models, and clinical data, providing a multidisciplinary perspective on efflux-associated resistance and virulence. The workflow of the literature search and selection strategy is summarized in Fig. 1.

## EPs in Antimicrobial and Antitumoral Resistance

### Classification and mechanisms of EPs

Efflux pumps can extrude a wide variety of antimicrobial drugs used against bacteria, fungi, and other pathogens, contributing to reduced efficacy and multidrug resistance. In subsequent sections, we also address their role in extruding antitumor agents. Efflux pumps are membrane transport proteins that extrude compounds from the intracellular environment, contributing significantly to multidrug resistance across a wide range of organisms. These systems are categorized into six major families based on their structure and energy sources (Table 1):

**Table 1:** EP families: Energy sources, mechanisms, and role in antimicrobial resistance. The main families of efflux pumps (EPs), detailing their energy sources, transport mechanisms, representative organisms, and clinical relevance. It highlights the diversity of EP systems involved in multidrug resistance and their significance in human health, including bacterial infections and cancer.

EP family	Energy source	Mechanism	Representative organisms	Clinical relevance
ABC	ATP hydrolysis	Primary active transport via ATP-binding domains	Humans*, Mycobacteria, Staphylococcus aureus*	Antitumoral drug resistance, tuberculosis
MFS	Proton motive force	Secondary active transport using proton gradient	*Escherichia coli, Mycobacteria, Staphylococcus aureus*	Resistance to tetracyclines, macrolides, fluoroquinolones
RND	Proton motive force	Tripartite system spanning membranes, proton-driven	*Pseudomonas aeruginosa, E. coli, Acinetobacter baumannii*	MDR in Gram-negatives; major clinical concern
SMR	Proton motive force	Small, single-component pumps; proton-driven	*Staphylococcus aureus, Enterococcus spp.*	Contributes to resistance in Gram-positives
MATE	Sodium or proton gradient	Na+/H+ antiporters for extrusion of toxic compounds	*Vibrio cholerae, E. coli*	Efflux of fluoroquinolones, aminoglycosides
PACE	Proton motive force	Efflux of biocides and antiseptics; newly described	*Acinetobacter baumannii, Klebsiellapneumoniae*	Resistance to antiseptics and disinfectants

• The ATP-binding cassette (ABC) family uses ATP hydrolysis for active transport.
• The major facilitator superfamily (MFS), small multidrug resistance (SMR) family, and resistance-nodulation-division (RND) family depend on the proton motive force.
• The multidrug and toxic compound extrusion (MATE) family utilizes sodium or proton gradients.
• The most recently identified, proteobacterial antimicrobial compound efflux (PACE) family.

Importantly, ABC transporters represent a converging theme across domains: in bacteria, they extrude a wide variety of antimicrobials, while in cancer cells, they mediate resistance to chemotherapeutics such as taxanes and topoisomerase inhibitors. This dual role emphasizes the translational relevance of efflux inhibition strategies.

### Efflux and antimicrobial resistance

EPs contribute to both intrinsic and acquired antimicrobial resistance (AMR) by reducing intracellular drug accumulation, allowing microorganisms to survive in the presence of different compounds. This resistance mechanism is particularly complex due to the remarkable diversity and redundancy among efflux systems. While some EPs, like the MFS transporter TetA, show narrow specificity (*e.g.*, tetracyclines), others, such as the RND system AcrAB-TolC in *E. coli* are highly promiscuous, expelling fluoroquinolones, β-lactams, aminoglycosides, macrolides, detergents, and dyes (Wright, 2025).

Efflux pumps are widespread among bacteria and can actively extrude structurally diverse antimicrobial agents. This broad substrate range plays a central role in driving multidrug resistance in both Gram-negative and Gram-positive pathogens.

One of the earliest examples was the identification of tetracycline efflux systems in *E. coli* in the 1980s (McMurry & Levy, 1978; McMurry, Petrucci Jr & Levy, 1980). In Gram-negative bacteria, the combination of EPs and the outer membrane barrier drastically limit drug action. Overexpression of efflux pumps lowers intracellular drug concentrations to subtherapeutic levels, reducing antimicrobial efficacy and promoting treatment failure. This mechanism, first proposed by Levy (1992), was later confirmed across multiple microorganisms (Piddock, 2006; Blair et al., 2015).

In Gram-negative bacteria, efflux pumps belonging to the RND family are major contributors to multidrug resistance. A classic example is the AcrAB-TolC system in *Escherichia coli* and *Salmonella enterica*, which exports β-lactams, tetracyclines, fluoroquinolones, and several other antimicrobials air (Blair et al., 2015; Nishino et al., 2021). Other clinically relevant Gram-negative efflux systems include MexAB-OprM in *Pseudomonas aeruginosa* and AdeABC in *Acinetobacter baumannii* (Magnet, Courvalin & Lambert, 2001; Coyne, Courvalin & Perichon, 2011). In Gram-positive bacteria, multidrug resistance is largely associated with members of the MFS and ABC families. For instance, the NorA pump in *Staphylococcus aureus* and PmrA in *Streptococcus pneumoniae* mediate resistance to fluoroquinolones and macrolides, respectively (Poole, 2001; Kaatz, McAleese & Seo, 2005; Costa et al., 2011). Together, these systems exemplify how efflux pumps in both Gram-negative and Gram-positive pathogens contribute to therapeutic failure.

In mycobacteria, efflux-mediated resistance was first reported in the mid-1990s. Liu, Takiff & Nikaido (1996) identified LfrA, an MFS transporter in *Mycobacterium smegmatis*, conferring resistance to fluoroquinolones and serving as a model for studies of efflux in *M. tuberculosis*. This led to the discovery of Tap by Ainsa et al. (1998), which mediates resistance to tetracycline and other agents.

Subsequent studies in *M. tuberculosis* and *M. bovis* have identified multiple efflux systems:

• De Rossi et al. (2002) reported 16 putative MFS-encoded transporters
• Silva et al. (2001) characterized P55, implicated in resistance to aminoglycosides, tetracyclines, and macrolides.
• da Silva et al. (2011) highlighted the high number of efflux-related genes in *M. tuberculosis*, relative to its genome size, emphasizing their relevance in both intrinsic and acquired resistance.

Further studies revealed the importance of RND-type pumps, such as the MmpL family. Notably, MmpL5 is involved in resistance to bedaquiline and clofazimine, while Rv0678 regulates the MmpS5-MmpL5 operon (Andries et al., 2014; Villellas et al., 2017). Interesting, these systems also contribute to bacterial persistence in macrophages (Rodrigues et al., 2013).

Although bacterial efflux has been more extensively characterized, fungal pathogens also deploy highly conserved transporters that significantly contribute to drug resistance, particularly against azole-class antifungals. *Candida albicans* expresses ABC transporters such as Cdr1 and Cdr2, as well as the MFS-type Mdr1, which collectively contribute to fluconazole resistance and virulence (Coleman & Mylonakis, 2009; Sanguinetti, Posteraro & Lass-Florl, 2015). Similar mechanisms are observed in *Aspergillus fumigatus*, where transporters such as AtrF and AfuMDR4 are implicated in reduced susceptibility to triazoles (Meneau, Coste & Sanglard, 2016).

These EPs are active in both planktonic cells and biofilms, where they play a critical role in collective protection against antifungal agents. In *Candida auris* the overexpression of efflux pumps plays a critical role in azole resistance and therapeutic evasion by actively reducing intracellular drug concentrations, thereby compromising treatment efficacy (Zavrel & White, 2015; Rybak et al., 2019).

### Efflux and antitumoral resistance

The MDR in cancer cells has been widely investigated since the 1970s, when early studies in Chinese hamster cells revealed cross-resistance to unrelated chemotherapeutic agents such as vinblastine, vincristine, and daunomycin (Biedler & Riehm, 1970; Dano, 1973). These findings pointed to a shared mechanism of resistance, which was later confirmed by the identification of P-glycoprotein (P-gp)—the first ATP-binding cassette (ABC) transporter associated with drug efflux in tumor cells (Juliano & Ling, 1976).

In cancer, EPs of the ABC superfamily are central to treatment failure. The most clinically relevant include:

• P-glycoprotein (P-gp/ABCB1**)**: Overexpressed in multiple cancers such as acute myeloid leukemia, gliomas, hepatocellular carcinoma, and small cell lung cancer, P-gp reduces intracellular concentrations of chemotherapeutic agents, including paclitaxel, doxorubicin, and vincristine. This efflux limits drug efficacy and is associated with poor clinical outcomes (Robey et al., 2018; Zhang et al., 2015).
• Breast cancer resistance protein (BCRP/ABCG2): BCRP extrudes drugs such as mitoxantrone, topotecan, and SN-38. Its overexpression contributes to resistance in breast cancer, acute lymphoblastic leukemia, and gastrointestinal tumors (Nakanishi & Ross, 2012).
• Multidrug resistance-associated proteins (MRPs/ABCC family): MRP1 mediates resistance to anthracyclines, vinca alkaloids, methotrexate, and some tyrosine kinase inhibitors, being frequently overexpressed in lung cancer, neuroblastoma, and pancreatic cancer (Cole, 2014).

Conditions such as hypoxia, low pH, and inflammation can upregulate EP expression or alter drug bioavailability, exacerbating treatment resistance (Robey et al., 2018). Beyond drug extrusion, MDR often results from complex crosstalk between efflux and other resistance-related pathways, including DNA repair mechanisms, evasion of apoptosis, and metabolic reprogramming. For instance, overexpression of ABC transporters such as P-gp and BCRP has been associated with upregulation of DNA repair proteins (*e.g.*, BRCA1/2), altered expression of apoptotic regulators (*e.g.*, Bcl-2, caspases), and increased glycolytic activity that sustains tumor cell survival under therapeutic stress (Gottesman, Fojo & Bates, 2002). This networked resistance phenotype complicates single-agent therapy and underscores the need for combination strategies that simultaneously target efflux activity and complementary pathways (Fletcher et al., 2010; Holohan et al., 2013; Kathawala et al., 2015).

## Biofilms, Virulence in Bacterial Pathogens and the Role of EPs in Microbial Communities

Efflux not only contribute to AMR but also play a pivotal role in the virulence of bacterial and fungal pathogens (Table 2). Efflux enhances adhesion, invasion, and biofilm formation, enabling microbes to evade host immune and establish persistent infections (Alcalde-Rico et al., 2016; Van Acker & Coenye, 2017; Martinez et al., 2009). The dual role of efflux as mediator of both drug resistance and virulence, places them at the center of microbial pathogenesis. By exporting toxic host-derived compounds, such as antimicrobial peptides and reactive oxygen species, so EPs help pathogens survive in hostile environments and colonize host tissues (Piddock, 2006; Poole, 2007; Blair et al., 2015; Du Toit, 2017).

Biofilms are structured microbial communities embedded in an extracellular matrix composed of polysaccharides, proteins, and nucleic acids, which provides mechanical stability and protection against antimicrobials and host defenses, making these infections difficult to eradicate. EPs play a central role in biofilm formation, maintenance, and persistence by exporting signaling molecules such as quorum-sensing autoinducers, metabolic byproducts, and virulence factors, thereby regulating surface adhesion and matrix production (Flemming et al., 2016; Van Acker & Coenye, 2017). This multifunctional activity enables pathogens to thrive in diverse environments and poses a major challenge to antimicrobial therapy. Therefore, targeting EP activity within biofilms is a promising strategy to disrupt these resilient communities and enhance treatment outcomes.

EPs of the RND and MFS families play multifaceted roles in bacterial virulence by modulating quorum sensing, toxin secretion, biofilm development, and metabolic adaptation. In *P. aeruginosa*. MexAB-OprM exports quorum-sensing molecules such as N-acyl homoserine lactones, which coordinate biofilm development (Blanco et al., 2016). Disruption of MexAB-OprM has been shown to impair biofilm formation and reduce virulence in Li & Nikaido (2009). Other EPs, including MexCD-OprJ and MexEF-OprN, contribute to the secretion of virulence factors like pyocyanin, a redox-active toxin involved in tissue damage and immune modulation (Linares et al., 2005; Olivares Pacheco et al., 2017; Behzadi et al., 2022).

**Table 2:** Multifunctional roles of EPs in microorganisms. Diverse biological roles of efflux pumps (EPs) beyond antimicrobial resistance, including virulence factor secretion, immune evasion, and environmental detoxification. It provides examples of microbial groups, associated efflux systems, exported substrates, and their respective biological impacts.

Function	Microbial groups	Efflux systems	Exported substrates	Biological impact
Antimicrobial resistance	Gram-positive, Gram-negative, Mycobacteria, Fungi	NorA, MexAB-OprM, AcrAB-TolC, MmpL, Cdr1/Cdr2	Antimicrobials (fluoroquinolones, azoles, rifampicin)	Drug efflux leading to treatment failure
Virulence factor secretion	*P. aeruginosa, E. coli*)	MexAB-OprM, AcrAB-TolC	Toxins (elastase, exotoxin A, hemolysin), adhesins, proteases	Tissue damage, immune suppression, colonization
Immune Evasion	*S. aureus, P. aeruginosa, M. tuberculosis, Candida* spp.	MprF, MexAB-OprM, MmpL, Cdr1/Cdr2	AMPs, ROS, mycolic acids, PDIMs, mycotoxins	Phagosome escape, cytokine suppression, intracellular survival
Environmental detoxification	Soil and water bacteria (*E. coli*, *P. aeruginosa*)	AcrAB-TolC, MexAB-OprM	PAHs, pesticides, heavy metals, solvents	Survival in polluted environments, ecological resilience

The NorA EP in *S. aureus*, a member of the MFS, is well recognized for mediating fluoroquinolone resistance. Beyond aAMR, NorA plays a critical role in biofilm formation by exporting autoinducers and toxic metabolites that stabilize the community structure, enhancing survival on surfaces and within host tissues, offering protection against antimicrobials and host immune responses (Sabatini et al., 2017). Its overexpression has been linked to persistent infections, particularly in association with indwelling medical devices (Costa et al., 2013; Sinha, Aggarwal & Singh, 2024).

Similarly, overexpression of AdeFGH in *Acinetobacter baumannii*, enhances biofilm formation, promotes surface attachment, and supports bacterial survival (He et al., 2015). These characteristics facilitate colonization of hospital environments and the establishment of chronic infections, particularly among immunocompromised patients.

In *M. tuberculosis*, efflux systems belonging to the RND family, notably the membrane proteins large (MmpL) and mycobacterial small membrane protein (MmpS), such as MmpL7 and the MmpL5, are involved in the export of lipid virulence factors and AMR. These transporters support bacterial survival within macrophages by modulating the cell wall composition and detoxifying the intracellular environment. Notably, MmpL7 mediates the transport of phthiocerol dimycocerosates, complex lipids that enhance virulence by interfering with host immune responses. Overexpression of MmpL5-MmpS5 complex, is the first form of tolerance towards antimicrobials, underscoring their dual role in pathogenesis and drug resistance. Later, during the long treatment exposure, mutations in the Rv0678 gene (MmpS5-MmpL5 transcriptional repressor), the upregulation becomes stable, and this overexpression contributes to resistance against to bedaquiline and clofazimine, causing limitations to implementation and success of the new therapeutic regimens for TB treatment (Kaniga et al., 2022; Pasca et al., 2005; Machado et al., 2016).

### EPs and virulence in fungal pathogens

In addition to their well-established role in antifungal resistance, EPs in fungal pathogens also contribute to virulence by mediating toxin secretion, biofilm formation, and survival within host environments. In *Candida albicans*, Cdr1 and Cdr2, and Mdr1, have been linked to azole resistance and virulence traits, including increased adhesion, tissue invasion, and biofilm development (Holmes et al., 2008; Prasad & Rawal, 2014). Overexpression of these EPs enhances fungal persistence under host-imposed stress, such as exposure to antimicrobial peptides and oxidative damage. Moreover, in *A. fumigatus*, MFS-type transporters encoded within secondary metabolite gene clusters are implicated in the export of mycotoxins—compounds that facilitate host damage and immune evasion (Del Sorbo, Schoonbeek & De Waard, 2000; Keller, 2019). These transporters are thought to be activated in response to toxin accumulation, enabling rapid secretion and reducing intracellular toxicity. In both yeasts and filamentous fungi (LaFleur, Kumamoto & Lewis, 2006; Cannon et al., 2009).

## Classical Detection Strategies of Efflux and Methodological Limitations

Several experimental approaches have been employed to assess efflux activity, each with inherent strengths and drawbacks:

### Detection efflux by EP inhibitors

Compounds like phenylalanine-arginine β naphthylamide (PAβN) and carbonyl cyanide m-chlorophenylhydrazone (CCCP) are used alongside antimicrobials to evaluate efflux participation. A significant reduction in the minimum inhibitory concentration (MIC) in the presence of an EP inhibitor (EI) is indicative of efflux involvement (Lomovskaya et al., 2001). However, EIs may exert off-target effects and lack specificity, complicating data interpretation (Pages, Amaral & Fanning, 2011).

### Fluorometric assays

These assays utilize fluorescent dyes—such as ethidium bromide and Hoechst 33342—that are actively expelled by EPs. Reduced intracellular fluorescence in the presence of these dyes suggests high efflux activity. Fluorometric methods offer real-time, quantitative monitoring and are highly sensitive, making them essential tools for evaluating pump function and inhibitor efficacy (Martins et al., 2011). Confounding variables such as dye toxicity, uptake variability, and non-specific binding can impair result accuracy. Recent innovations, such as more stable fluorescent probes and live-cell imaging techniques, have improved reliability and broadened applicability (Paixao et al., 2009).

### Molecular approaches

Molecular approaches are also used for characterizing efflux-mediated resistance. Targeted gene inactivation, using methods such as homologous recombination or CRISPR-Cas9 (clustered regularly interspaced short palindromic repeats/CRISPR-associated protein 9), allows the functional validation of specific efflux systems by observing changes in susceptibility after gene disruption Additionally, transcriptomic analyses, particularly RNA sequencing enable the assessment of efflux gene expression under various conditions, revealing regulatory responses to antimicrobial exposure. Comparative transcriptomics between wild-type and efflux-deficient strains has been instrumental in identifying induced efflux systems and co-regulated resistance pathways. Together, these strategies provide a comprehensive understanding of efflux mechanisms and support the development of novel therapeutic interventions (Li, Plesiat & Nikaido, 2015; Li et al., 2018).

## Emerging Technologies to Detection of Efflux

Recent advances in technology have led to the development of more sophisticated methods for detecting efflux-mediated resistance, offering new tools to address the challenges posed by MDR pathogens. These emerging technologies not only improve the accuracy and efficiency of EP detection but also provide novel insights into the mechanisms of resistance and potential therapeutic targets.

### Microfluidics and single-cell analysis

Microfluidic platforms and single-cell analysis techniques have revolutionized the study of EPs by enabling real-time monitoring of efflux activity at the single-cell level. These methods allow to observe the heterogeneity within bacterial populations, where individual cells may exhibit varying levels of efflux activity due to genetic or environmental factors (Matsumoto et al., 2011). This approach has revealed that even genetically identical cells can display significant differences in efflux activity, which may contribute to the survival of subpopulations under antibiotic pressure (Iino et al., 2012). Single-cell analysis also provides insights into the dynamics of EP regulation and the role of stochastic gene expression in resistance development (Blair & Piddock, 2016).

### Biosensors

Genetically engineered biosensors are being developed to detect efflux activity with high specificity and sensitivity. These biosensors typically consist of reporter genes, such as fluorescent or luminescent markers, that are activated in response to EP function. A small molecule-responsive biosensor was developed to link PDR gene activation to growth in *Saccharomyces cerevisiae*. Using diverse inducers and a homozygous diploid deletion library, genome-wide screens identified both known and novel regulators of PDR. Notably, genes involved in the mitotic spindle assembly checkpoint were found to modulate EP expression, revealing a new connection between PDR and cell cycle control. Biosensors offer several advantages, including rapid detection, high throughput, and the ability to monitor efflux activity in live cells without disrupting their physiology (Li et al., 2019).

### Advanced imaging techniques

Techniques such as super-resolution microscopy and cryo-electron microscopy (cryo-EM) are providing unprecedented insights into the structure and function of EPs. For example, cryo-EM has been used to visualize the three-dimensional structure of the AcrAB-TolC complex in *E. coli*, revealing how the pump undergoes conformational changes to transport substrates across the bacterial membrane (Du et al., 2018; Klenotic, Morgan & Yu, 2021). These structural insights are critical for the rational design of EIs that can block EP function without affecting essential cellular processes.

In summary, emerging technologies are transforming the study of efflux-mediated resistance, offering new opportunities to understand the mechanisms of efflux develop targeted therapies, and combat the global threat of multidrug-resistant infections. However, their successful implementation will require interdisciplinary collaboration, investment in infrastructure, and the integration of experimental and computational approaches.

## Efflux Inhibitors

### Mechanisms and potential candidates

A variety of strategies have been proposed to inhibit drug efflux and restore antimicrobial efficacy. These include direct inhibition of EPs by competitive or non-competitive EIs, disruption of EP assembly, and interference with the proton motive force that drives secondary active transporters. Other approaches focus on the transcriptional regulation of efflux genes, aiming to suppress pump expression. Additionally, structural modification of antimicrobial agents to evade efflux recognition, or trapping transporters in inactive conformations, represents promising complementary tactics. EP inhibitors, especially when used as adjuvants, offer a powerful means to restore the effectiveness of existing antimicrobials and delay the emergence of resistance, reinforcing the therapeutic value of targeting efflux as part of a comprehensive antimicrobial strategy. The mechanism more frequently described is competitive inhibition, where EIs bind directly to the substrate-binding site of the EP, preventing the actual substrate from accessing and being extruded. This direct competition effectively reduces the efflux of antimicrobial agents, increasing their intracellular concentrations, and if used as an adjuvant with an antimicrobial, will reduce its effective dose. For instance, certain inhibitors have been shown to occupy the substrate-binding pocket of the AcrB EP in *E. coli*, thereby blocking drug efflux (Kim et al., 2021b).

Disruption of EP assembly was showed through EIs bind to AcrA, a component of the prototypical AcrAB-TolC pump, change its structure *in vivo*, inhibit efflux of fluorescent probes, and potentiate the activities of antimicrobials in *E. coli* and other Gram-negative bacteria (Abdali et al., 2017).

Another strategy involves allosteric modulation, wherein EIs bind to sites distinct from the substrate-binding region, inducing conformational changes that impair the pump’s function. This alteration can decrease the pump’s affinity for its substrates or disrupt the conformational changes necessary for substrate transport. Recent studies have identified pyridylpiperazine-based compounds that act as allosteric inhibitors of RND-type multidrug EPs, effectively hindering their activity (Zgurskaya et al., 2021).

Energy disruption represents another approach, targeting the energy sources essential for pump operation. EPs often rely on the proton motive force or ATP hydrolysis to actively transport substrates across the membrane, EIs can effectively inhibit efflux activity (Sharma, Gupta & Pathania, 2019; Li et al., 2024).

It was identified that both synthetic and natural EIs with improved efficacy and specificity have been reported, mainly in microbial models. However, EIs are also being actively investigated in oncology, with several candidates—such as tariquidar, Ko143, and SCO-101—under evaluation for their ability to reverse multidrug resistance in cancer cells, including in ongoing clinical trials. For instance, MBX-2319, a synthetic pyranopyridine derivative, targets the AcrAB-TolC system in *E. coli*, enhancing the bactericidal activity of ciprofloxacin (Opperman et al., 2014). Similarly, natural compounds such as baicalein have been found to inhibit EPs in *S. aureus*, restoring the effectiveness of antimicrobial agents (Mao et al., 2023).

An alternative strategy to inhibit EP activity involves repressing the expression of corresponding genes. This approach employs antisense oligonucleotides or small interfering RNA (siRNA) to selectively prevent the transcription or translation of genes encoding EPs (Kong et al., 2015). Further, some efflux inhibitors, including tariquidar and verapamil, have been studied in both microbial and oncological contexts, highlighting the overlap between antimicrobial resistance and cancer multidrug resistance. This convergence suggests that lessons learned in one field may inform the other, with microbial models providing mechanistic insights and oncology trials offering translational evidence for safety and efficacy. Such cross-domain perspectives reinforce the potential of efflux inhibition as a unifying therapeutic strategy.

### CRISPR/Cas9-Mediated EP modulation: a novel strategy against multidrug resistance

The CRISPR/Cas9 system is a gene-editing technology derived from a bacterial adaptive immune mechanism. Originally used by bacteria and archaea to defend against invading genetic elements, this system has been adapted as a powerful and precise tool for genome editing in eukaryotic and prokaryotic cells (Vaghari-Tabari et al., 2022). CRISPR/Cas9 enables targeted modification, disruption, or regulation of specific genes, and has been increasingly explored as a strategy to combat antimicrobial resistance by interfering with EP expression and function.

Collectively, these diverse strategies underscore the potential of EIs to combat MDR by targeting efflux mechanisms through competitive inhibition, allosteric modulation, energy disruption, and gene expression. Recent studies have demonstrated that CRISPR/Cas9-mediated disruption of these efflux transporter genes can restore antimicrobial and antitumoral activity (Vaghari-Tabari et al., 2022; Sajid, Rahman & Ambudkar, 2023).

In bacteria, CRISPR interference (CRISPRi)—a variant of CRISPR/Cas9 that represses transcription without DNA cleavage—has been used to silence EP genes. Wan et al. (2022) developed a CRISPRi system targeting the AcrAB-TolC pump *in E. coli,* resulting in substantial downregulation of efflux gene expression and increased susceptibility to multiple antibiotics, including rifampicin, erythromycin, and tetracycline. Additionally, this system reduced biofilm formation, further enhancing antimicrobial efficacy.

More broadly, CRISPR/Cas systems have been included among non-traditional strategies to overcome antimicrobial resistance. Their integration into combinatorial therapies, together with antimicrobial peptides, phage therapy, and EP inhibitors has opened new avenues for overcoming resistance in both clinical and experimental settings (Konwar et al., 2022).

### Natural products as EIs candidates

Natural products have emerged as a promising source of EIs due to their structural diversity and potential for synergistic effects with existing antimicrobials. For example, 5′-methoxyhydnocarpin-D is a flavonolignan produced by species like *Berberis fremontii, Berberis repens,* and *Berberis aquifolia* that can enhance the antibacterial activity of ciprofloxacin and tobramycin against *S. aureus* (Gaurav et al., 2023). Similarly, curcumin, a polyphenol from turmeric (*Curcuma longa*), has demonstrated to inhibit EPs in *P. aeruginosa*, reducing resistance to ciprofloxacin (Negi et al., 2014).

Baicalein is a flavonoid from *Scutellaria baicalensis* was recently described inhibitor EPs in *S. aureus*, reducing resistance to fluoroquinolones (Moulick & Roy, 2024). Carvacrol and Thymol are phenolics compounds found in *Origanum vulgare* and *Thymus vulgaris* inhibits NorA EP in MDR *S. aureus* strains (Dos Santos Barbosa et al., 2021).

Reserpine is an alkaloid obtained from *Rauvolfia serpentina* described almost 30 years ago, inhibits EPs in Gram-positive bacteria, including *S. aureus*, *S. pneumoniae* and *M. tuberculosis* (Angelini, 2024; Schmitz et al., 1998; Garvey et al., 2011; Huang et al., 2013). Plumbagin is a naturally occurring napthoquinone, obtained from *Plumbago zeylanica* is known to modulate cellular proliferation, carcinogenesis, and radio-resistance and is also a cardiotonic, hepatoprotective, and neuroprotective agent. Also, has been shown to inhibit ABCG2 EP that is one of the reasons for the development of MDR in cancer (Shukla et al., 2007).

Microbial metabolites also have emerged as a promising source of EIs**,** Ethyl 4-bromopyrrole-2-carboxylate from the soil bacterium *Streptomyces* sp. demonstrates efficacy of the antibiotic-EI combination against *P. aeruginosa.* An indole (RP2) isolated from the soil bacterium has showed potential of EI in resistance in *S. aureus* (Tambat et al., 2019; Tambat et al., 2022).

### Synthetic compounds as EIs

Several synthetic compounds have been evaluated as EIs, with varying degrees of success. For example, MC-207,110 (also known as PaβN: Phe-Arg β-naphthylamide) is one of the earliest synthetic EIs studied. It has shown efficacy in inhibiting RND-type EPs in *P. aeruginosa* and *E. coli*, reversing resistance to fluoroquinolones and β-lactams (Lomovskaya et al., 2001). However, its clinical use is limited due to toxicity and off-target effects. Another notable synthetic EI is MBX2319, a pyridopyrimidine derivative that specifically targets the AcrAB-TolC efflux system in *E. coli* and other Enterobacteriaceae bacteria. MBX2319 has shown promise in preclinical studies, significantly reducing the MIC of antibiotics like ciprofloxacin and levofloxacin (Opperman et al., 2014). However, it has not yet advanced to clinical trials.

D13-9001 is an EI that targets the MexAB-OprM efflux system in *P. aeruginosa* which enhances the activity of β-lactams and fluoroquinolones. This compound has demonstrated potent efflux inhibitory activity *in vitro* and animal models, but its clinical development has been hindered by challenges related to pharmacokinetics and toxicity (Nakashima et al., 2013). A novel EI MBX-4191that targets the AcrAB-TolC system in *E. coli,* restored susceptibility to multiple antimicrobials (Vargiu et al., 2014). Tetrahydropyridines can act as a potential pharmacophore candidate with potential *in vitro* and *in silico* as inhibitor of efflux in *E. coli and M. abscessus* (Silva Jr et al., 2017; Vianna et al., 2019).

Pyrvinium, an anthelmintic traditionally used to treat pinworm infections, has shown promising antimicrobial properties. It was found to be effective against preformed biofilms and demonstrated excellent *in vivo* efficacy. When combined with ciprofloxacin, pyrvinium significantly enhanced activity against multidrug-resistant *S. aureus*, even at clinically achievable concentrations. This synergistic interaction suggests that the pyrvinium–ciprofloxacin combination could be a valuable strategy for treating persistent and biofilm-associated infections caused by MDR pathogens (Mahey et al., 2021). Nilotinib, a tyrosine kinase inhibitor approved for the treatment of chronic myeloid leukemia, has also emerged as a potential EP inhibitor, was shown to specifically inhibit the NorA EP in *S. aureus* when used in combination with ciprofloxacin. This inhibition significantly enhanced the antimicrobial activity of ciprofloxacin, particularly in the context of biofilms (Zimmermann et al., 2019).

A summary of representative natural and synthetic efflux inhibitors, their main targets, and contexts of use is provided in Table S1.

## Recent Advances in EI Research and Therapeutic Strategies

Further innovations in combating efflux-mediated resistance include gene editing and combination therapy approaches. A recent study introduced an inducible CRISPR interference (CRISPRi) system designed to silence components of the AcrAB-TolC EP in *E. coli*, aiming to curb the development of MDR. By targeting the acrA, acrB, and tolC genes using specific single-guide RNAs (sgRNAs), the system effectively reduced the expression of these key efflux components. The downregulation of the AcrAB-TolC complex led to enhanced susceptibility to multiple antibiotics and a noticeable reduction in biofilm formation (Wan et al., 2022).

Combination therapies, involving the co-administration of EIs with existing antimicrobials or chemotherapeutic agents, have shown promise in increasing intracellular drug concentrations and reversing multidrug resistance (Zhang, Wang & Ahn, 2023). Notably, next-generation EIs, such as tariquidar (a P-gp inhibitor) and Ko143 (a BCRP inhibitor), have demonstrated potential in restoring the efficacy of chemotherapeutic regimens, offering a new avenue for overcoming treatment failures in resistant cancer cells (Weidner et al., 2016; Lustig et al., 2022).

These advancements collectively represent a multi-faceted approach to mitigating efflux-mediated drug resistance, integrating natural and synthetic inhibitors, nanotechnology-driven drug delivery, genetic modulation, and strategic drug combinations to enhance treatment outcomes (Xue & Liang, 2012).

## Clinical Trials of EP Inhibitors (EIs)

Despite promising preclinical results, few EIs have hitherto progressed to clinical trials, mainly due to challenges such as toxicity, pharmacokinetic limitations, and the inherent complexity of efflux systems (Sharma, Gupta & Pathania, 2019). Only a limited number of EIs have advanced to clinical evaluation, offering valuable insights into their therapeutic potential for treating multidrug-resistant infections and chemotherapy-resistant cancers. Verapamil, a calcium channel blocker traditionally used for cardiovascular conditions, has been repurposed as an EI due to its ability to inhibit EPs in *M. tuberculosis* (Gupta et al., 2013). Verapamil acts by disrupting bacterial membrane function, inhibiting Ca^2^^+^ channels and ABC transporters, downregulating efflux pump genes, increasing intracellular drug accumulation, reducing MICs and FICI values, and improving systemic exposure to drugs without affecting bioavailability. Co-administration of verapamil has been shown in preclinical studies to enhance the activity of bedaquiline against *M. tuberculosis*, including drug-resistant strains, both *in vitro* and in murine models. This combination not only enhances early bactericidal activity but also helps prevent the emergence of resistant mutants. Moreover, verapamil may contribute to reducing drug tolerance and offer potential cardioprotective effects, countering the QTc prolongation associated with bedaquiline (Srikrishna et al., 2015). Recent clinical trial suggest that a higher dosage of verapamil can be safely used as an adjunctive treatment in rifampin-containing treatment regimens (Padmapriyadarsini et al., 2024).

SCO-101, a repurpose drug as well as verapamil, has shown preclinical efficacy in reversing resistance to irinotecan and taxanes. A phase II clinical trial (NCT04247256) is evaluating the safety, toxicity, and efficacy of combining SCO-101—an oral efflux inhibitor currently under investigation—with FOLFIRI, a standard chemotherapy regimen consisting of 5-fluorouracil, leucovorin, and irinotecan (a topoisomerase I inhibitor widely used in colorectal cancer). In addition, efflux inhibition has been investigated in combination with taxanes (such as paclitaxel and docetaxel), a class of drugs that stabilize microtubules and are used in multiple solid tumors (Ladekarl et al., 2023).

## Future Challenges and Directions

Clinical translation remains challenging, as efflux pump inhibitors face limitations related to safety, pharmacokinetics, and redundancy of transport systems, together with the lack of rapid diagnostics to identify efflux-driven resistance in patients (Sharma, Gupta & Pathania, 2019). Examples illustrate these hurdles: in tuberculosis, *Rv0678*-mediated regulation of MmpS5–MmpL5 reduces susceptibility to BPaLM regimens (Andries et al., 2014; Villellas et al., 2017; Kaniga et al., 2022) while verapamil has shown potential as an adjuvant but requires careful evaluation in clinical protocols (Gupta et al., 2013; Srikrishna et al., 2015; Padmapriyadarsini et al., 2024). In medical mycology, biofilm-associated efflux activity contributes to azole failure, making early combination therapy and source control the most pragmatic solutions (LaFleur, Kumamoto & Lewis, 2006; Coleman & Mylonakis, 2009; Sanguinetti, Posteraro & Lass-Florl, 2015; Meneau, Coste & Sanglard, 2016), and in oncology, overexpression of ABC transporters drives chemoresistance, with next-generation inhibitors such as tariquidar and SCO-101 showing promise when applied in biomarker-guided trials (Weidner et al., 2016; Lustig et al., 2022; Ladekarl et al., 2023). Overall, anti-efflux strategies are not stand-alone interventions but may add value when patient selection, PK/PD optimization, and rational trial design converge.

Efflux systems are central drivers of resistance in both infectious diseases and cancer, yet they remain a largely unmet challenge in therapeutic development. Although natural and synthetic efflux pump inhibitors (EIs) have been proposed as promising solutions, their clinical translation has been severely hindered by issues of toxicity, lack of selectivity, suboptimal pharmacokinetics, and the intrinsic redundancy of efflux networks. Despite decades of research, few EIs have hitherto reached advanced clinical stages, highlighting a persistent gap between experimental findings and real-world applicability.

There are now compelling evidence that mutations in transcriptional repressor of the MmpS5-MmpL5 efflux pump system, lead to upregulation of this efflux mechanism, resulting in decreased susceptibility to the backbone of the new anti-MDR-TB regimen, BPaLM, emphasizing the challenges posed by such resistance mechanisms to the efficacy of this new WHO recommended therapeutic approach and the urgent need to develop therapeutic adjuvants to prevent it.

Emerging strategies, including computational drug design, nanoformulations, and combination therapies, offer hope but are still in their infancy and face significant hurdles regarding regulatory approval and scalability. Moreover, the multifunctionality of efflux pumps, implicated not only in drug resistance but also in bacterial virulence and environmental adaptation, complicates their therapeutic targeting without unintended ecological consequences.

Thus, while efflux inhibition remains an attractive concept, it demands a more realistic and integrative approach, prioritizing mechanistic understanding, careful validation in clinically relevant models, and the design of context-specific interventions. Without substantial interdisciplinary efforts and a reassessment of current strategies, the goal of effectively overcoming efflux-mediated resistance risks remaining aspirational rather than achievable.

##  Supplemental Information

10.7717/peerj.20360/supp-1 Supplemental Information 1

## Additional Information and Declarations

### Data Availability

The following information was supplied regarding data availability: This is a literature review.
