# Supplementary material for: Efflux systems driving resistance and virulence across biological domains
Source: PeerJ. 2025 Nov 26;13:e20360. doi: 10.7717/peerj.20360 (PMC12664331; doi:10.7717/peerj.20360)
Supplement: Supplemental Information 1 [file peerj-13-20360-s001.docx]

# Supplementary Table S1 – Representative natural and synthetic efflux inhibitors, their main targets, and contexts of use

| Inhibitor | Origin | Target/Family | Context of Use | Reference |
| --- | --- | --- | --- | --- |
| 5′-methoxyhydnocarpin-D | Natural (flavonolignan, Berberis) | NorA (S. aureus) | Potentiates ciprofloxacin and tobramycin | Gaurav et al., 2023 |
| Curcumin | Natural (polyphenol, Curcuma longa) | EPs of P. aeruginosa | Reduces ciprofloxacin resistance | Negi et al., 2014 |
| Baicalein | Natural flavonoid (Scutellaria baicalensis) | NorA (S. aureus) | Reduces fluoroquinolone resistance | Moulick & Roy, 2024 |
| Carvacrol / Thymol | Natural monoterpenes (oregano, thyme) | NorA (MDR S. aureus) | Inhibit fluoroquinolone resistance | Dos Santos Barbosa et al., 2021 |
| Reserpine | Natural alkaloid (Rauvolfia serpentina) | MFS, multiple Gram-positives | Inhibits efflux in S. aureus, S. pneumoniae, M. tuberculosis | Schmitz et al., 1998; Garvey et al., 2011 |
| Plumbagin | Natural (Plumbago zeylanica) | ABCG2 (cancer) | Blocks tumor MDR | Shukla et al., 2007 |
| Ethyl-4-bromopyrrole-2-carboxylate | Microbial metabolite (Streptomyces sp.) | EPs in P. aeruginosa | Potentiates antibiotics | Tambat et al., 2019/2022 |
| PaβN (MC-207,110) | Synthetic | RND (E. coli, P. aeruginosa) | Reverses resistance to fluoroquinolones and β-lactams (limited by toxicity) | Lomovskaya et al., 2001 |
| MBX-2319 | Synthetic (pyridopyrimidine) | AcrAB-TolC (E. coli, Enterobacteriaceae) | Potentiates ciprofloxacin/levofloxacin | Opperman et al., 2014 |
| D13-9001 | Synthetic | MexAB-OprM (P. aeruginosa) | Potentiates β-lactams/fluoroquinolones | Nakashima et al., 2013 |
| MBX-4191 | Synthetic | AcrAB-TolC (E. coli) | Restores susceptibility to antimicrobials | Vargiu et al., 2014 |
| Pyrvinium | Repurposed drug (anti-helminthic) | Biofilms of MDR S. aureus | Synergy with ciprofloxacin | Mahey et al., 2021 |
| Nilotinib | Repurposed drug (anticancer) | NorA (S. aureus) | Potentiates ciprofloxacin, biofilm inhibition | Zimmermann et al., 2019 |
| Verapamil | Repurposed drug (Ca2+ channel blocker) | M. tuberculosis EPs, ABC | Increases intracellular drug accumulation; oncology trials | Gupta et al., 2013 |
| Tariquidar | Synthetic / repurposed (oncology) | ABC (P-gp) | Reverses MDR in tumors (clinical trials) | Robey et al., 2018 |
| Ko143 | Synthetic | ABCG2 (BCRP) | Blocks efflux in cancer | Weidner et al., 2016 |
| SCO-101 | Synthetic (Phase II clinical trial) | ABC transporters | Combined with FOLFIRI in colorectal cancer | Ladekarl et al., 2023 |
